# Minimize the Xylitol Production in *Saccharomyces cerevisiae* by Balancing the Xylose Redox Metabolic Pathway

**DOI:** 10.3389/fbioe.2021.639595

**Published:** 2021-02-26

**Authors:** Yixuan Zhu, Jingtao Zhang, Lang Zhu, Zefang Jia, Qi Li, Wei Xiao, Limin Cao

**Affiliations:** ^1^Key Laboratory of Plant Gene Resources and Biotechnology for Carbon Reduction and Environmental Improvement, College of Life Sciences, Capital Normal University, Beijing, China; ^2^China Center of Industrial Culture Collection, Beijing, China; ^3^Department of Biochemistry, Microbiology and Immunology, University of Saskatchewan, Saskatoon, SK, Canada

**Keywords:** *Saccharomyces cerevisiae*, xylitol, xylose, promoter, expression

## Abstract

Xylose is the second most abundant sugar in lignocellulose, but it cannot be used as carbon source by budding yeast *Saccharomyces cerevisiae*. Rational promoter elements engineering approaches were taken for efficient xylose fermentation in budding yeast. Among promoters surveyed, *HXT7* exhibited the best performance. The *HXT7* promoter is suppressed in the presence of glucose and derepressed by xylose, making it a promising candidate to drive xylose metabolism. However, simple ectopic expression of both key xylose metabolic genes *XYL1* and *XYL2* by the *HXT7* promoter resulted in massive accumulation of the xylose metabolic byproduct xylitol. Through the *HXT7*-driven expression of a reported redox variant, *XYL1-K270R*, along with optimized expression of *XYL2* and the downstream pentose phosphate pathway genes, a balanced xylose metabolism toward ethanol formation was achieved. Fermented in a culture medium containing 50 g/L xylose as the sole carbon source, xylose is nearly consumed, with less than 3 g/L xylitol, and more than 16 g/L ethanol production. Hence, the combination of an inducible promoter and redox balance of the xylose utilization pathway is an attractive approach to optimizing fuel production from lignocellulose.

## Introduction

The world’s energy demand continues to increase, and clean energy is urgently needed to solve various problems including greenhouse effects and environmental pollution caused by petroleum and coal energy. Fuel ethanol produced by fermentation is a source of renewable energy and can significantly alleviate the fossil fuel shortage (Alam et al., 2019). *Saccharomyces cerevisiae* is a major organism used for efficient fermentation of glucose to produce ethanol (Baghel et al., 2015). Xylose is the second most abundant sugar in lignocellulose, but efficient xylose fermentation by microorganisms faces great challenge (Salusjärvi, 2006).

Although *S. cerevisiae* cannot utilize xylose naturally (Ma and Liu, 2010; Wang et al., 2019), it can use xylulose, an isomer of xylose, to produce ethanol, making it attractive to introduce xylose metabolism pathways derived from other microorganisms to *S. cerevisiae* (Zhang et al., 2012). One strategy is to introduce *XYL1* and *XYL2*, encoding NADPH-linked xylose reductase (XR), and NAD-linked xylitol dehydrogenase (XDH), respectively, from the xylose-fermenting yeast *Pichia pastoris* (Cao et al., 2014). Another method is to introduce *XI* encoding xylose isomerase from bacteria or fungi (Brat et al., 2009; Seike et al., 2019). A major challenge with the XR-XDH system is the imbalance of coenzymes, because XR preferentially uses NADPH over NADH, while XDH uses NAD^+^ as a cofactor (Weyda et al., 2014), which causes NADH accumulation during the fermentation process. NADH cannot be fully oxidized through the respiration process (Matsushika et al., 2009), which leads to the accumulation of the byproduct xylitol and ultimately reduces ethanol production. Therefore, to reduce xylitol and increase ethanol becomes a critical strategy in the xylose-to-ethanol fermentation. Previous reports employed protein engineering technology to modify the coenzyme specificity of XR and XDH to increase ethanol and reduce xylitol production. Some Xyl1 derivatives, such as K270R, K270M, and R276H, have positive effects on increasing ethanol production and reducing xylitol yield (Kostrzynska et al., 1998; Watanabe et al., 2007a, b; Hyun and Yeong, 2016); however, significant amount of xylitol still remained in our previously reported xylose fermentation systems even after introducing the above *XYL1* mutants (Sang et al., 2016).

In this study, we hypothesized that combination of the reported *XYL1* mutations and altered expression of these mutant alleles can further improve the redox balance during xylose fermentation, resulting in reduced xylitol accumulation. To this objective, we first evaluated available *XR*-related mutants for their ability to improve the redox balance during xylose fermentation. Next, we surveyed a panel of previously reported strong promoters to drive xylose metabolic pathway genes. The combination of two approaches allows us to optimize xylose fermentation toward reducing xylitol accumulation and increasing ethanol production, laying foundation for the subsequent scientific research and industrial development.

## Materials and Methods

### Yeast Strains and Culture Media

The *S. cerevisiae* strains used in this study are all haploids derived from the diploid industrial strain YC-DM (Angel Yeast, China). Yeast cells were grown in a YPD medium (10 g/L yeast extract, 20 g/L peptone, and 20 g/L glucose). The YPX medium replaces glucose with either 50 g/L xylose for the standard fermentation condition, or different amount of xylose as indicated.

### Plasmid Construction

General methods for plasmid construction and primer design were as previously described (Zhang et al., 2019). Construction of the parental plasmids pUC-GU-3X (Supplementary Figure 1) and pUC-GU-KR-E9 has been previously described (Xiong et al., 2011). To replace the constitutive promoters from *P_*ADH*__1_-XYL1* and *P_*PGK*__1_-XYL2* by *P_*HXT*__7_*, *P_*FBA*__1_*, or *P_*TEF*__1_*, corresponding forward and reverse primers were used to amplify the desired fragments from genomic DNA by PCR and cloned into pUC-GU-KR-E9. Oligonucleotides used in this study are listed in Supplementary Table 1 and the plasmids used in this study are given in Supplementary Table 2.

### Yeast Strain Creation

The integration plasmids used in this study carry a *KanMX* selectable marker gene and contain a fragment from the *HOG1* locus. Prior to the yeast transformation, these plasmids were digested with *Hpa*I, which linearizes the plasmid at the *HOG1* locus. The transformed yeast cells were spread on a YPD plate containing 170 μg/mL G418. After 2-day incubation, individual colonies were screened by genomic PCR for the anticipated chromosome integration. Yeast strains used in this study are listed in Supplementary Table 3.

### Xylose Fermentation Conditions

The recombinant yeast cells were cultured overnight in YPD at 30∘C with 200 rpm shaking, collected by centrifugation at 5,000 rpm, washed twice with sterile distilled water and then transferred to a flask containing 100 mL YPX liquid medium to make the initial OD_600_ = 0.2. Fermentation was carried out at 30∘C with 200 rpm shaking for 120 h, during which period 1 mL samples were retrieved at indicated time intervals for the measurement.

### Metabolite Analysis

To analyze yields of xylose, xylitol, and ethanol in the yeast culture, Aglient 1100 HPLC (Waters, Milford, United States) along with Aminex HPX 87H column (Bio-Rad, United States, mobile phase was 5 mM H_2_SO_4_) and Waters 2410 refractive index detector were used. The flow rate was 0.4 mL/min, the column temperature was 40∘C, and finally the substance concentration in the culture was obtained according to the integral value and the standard curve (Xiong et al., 2011).

## Results and Discussion

### Effects of Xylose Reductase Variants on the Efficient Utilization of Xylose

The wild-type *P. pastoris* xylose reductase Xyl1 preferentially uses NADPH as a cofactor, and the coenzyme affinity for Xyl1 can be altered through amino acid substitutions. In this study, we chose four previously reported Xyl1 variants, namely K270G, K270M, K270R/N272D, and K270R (Kostrzynska et al., 1998; Watanabe et al., 2007c; Xiong et al., 2013), and integrated each of them into the haploid strain derived from diploid YC-DM genome at the *HOG1* locus by homologous recombination to obtain four engineered strains E6 (K270G), E7 (K270M), E8 (K270R/N272D), and E9 (Xyl1-K270R). Meanwhile, strain D9 contains the wild-type *XYL1* gene integrated into the same locus to serve as a control. These five engineered strains were fermented in the YPX medium for 120 h to evaluate their effects on xylose metabolism.

**FIGURE 1:**
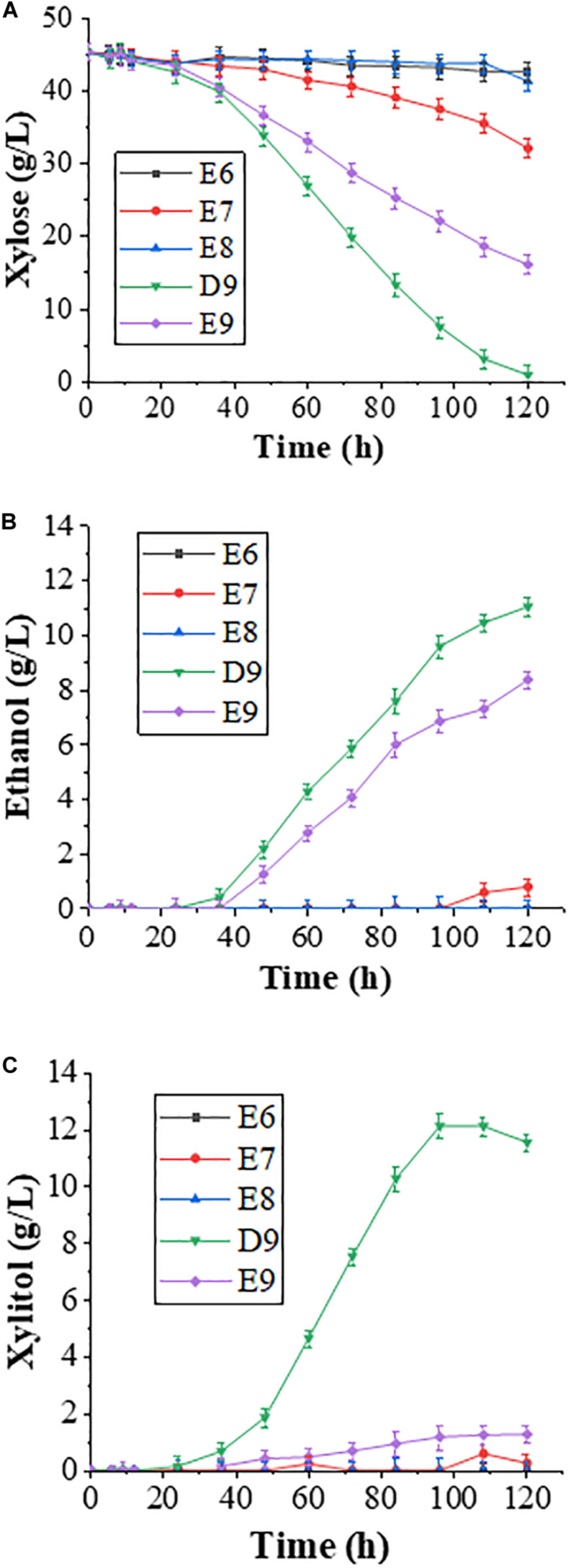
Fermentation results with E6, E7, E8, and E9 engineered strains in a YPX medium containing 50 g/L xylose. Samples were taken at different time points, and fermentation data were obtained through HPLC analysis. Time-dependent **(A)** xylose consumption; **(B)** ethanol production; and **(C)** xylitol accumulation. Error bars represent standard deviations from three independent experiments.

The fermentation results revealed that strains K270G, K270M, and K270R/N272D reduced xylose consumption (Figure 1A), and almost no ethanol production was observed (Figure 1B). Thus, these three Xyl1 variants were not further investigated in this study despite their previously reported positive effects in other yeast strains (Xiong et al., 2011, 2013). In contrast, strain E9 could effectively consume xylose, which is consistent with our previous observations (Xiong et al., 2013). From the perspective of xylose utilization efficiency, Xyl1 was more efficient than Xyl1-K270R. Within 120 h, the remaining xylose was less than 1 g/L in the D9 culture in comparison to about 16 g/L xylose in E9 (Figure 1A). It was speculated that Xyl1-K270R reduces the enzyme activity and hence cannot quickly utilize xylose. The xylitol byproduct was 11.54 and 1.26 g/L in D9 and E9 cultures, respectively (Figure 1C), suggesting a serious redox imbalance in D9, leading to the accumulation of xylitol. The above observations are consistent with our previous report, in which a strain carrying wild-type *XYL1* displays 20% faster xylose consumption and 2.5-fold higher xylitol yield than its isogenic *XYL1-K270R* strain (Zhang et al., 2018). It has been reported that the *XYL1-K270R* mutation results in a 31% reduction in unfavorable xylitol excretion (Watanabe et al., 2007b). In this study, the xylitol level was reduced by nearly 90% by the *XYL1-K270R* mutation, effectively solving the problem of xylitol accumulation during fermentation. Indeed, strain E9 produces lower xylitol yield than that of a reported strain TMB3270 under similar fermentation conditions (Bengtsson et al., 2009), suggesting that our metabolic engineering strategy is effective.

Although the ethanol production by D9 (11.03 g/L) was higher than that by E9 (8.36 g/L; Figure 1B), its increased xylitol yield may not be conducive to ethanol production. Overall, the two engineered strains differ greatly in xylose utilization and xylitol production, raising a possibility to further increase ethanol production by reducing xylitol accumulation. Since the xylitol production after introducing Xyl1-K270R is nearly tenfold lower than that of Xyl1 in this study, we wish to further improve xylose utilization based on Xyl1-K270R.

### Xylose Consumption and Xylitol Production Is Positively Correlated With the *XYL1* Promoter Strength

**FIGURE 2:**
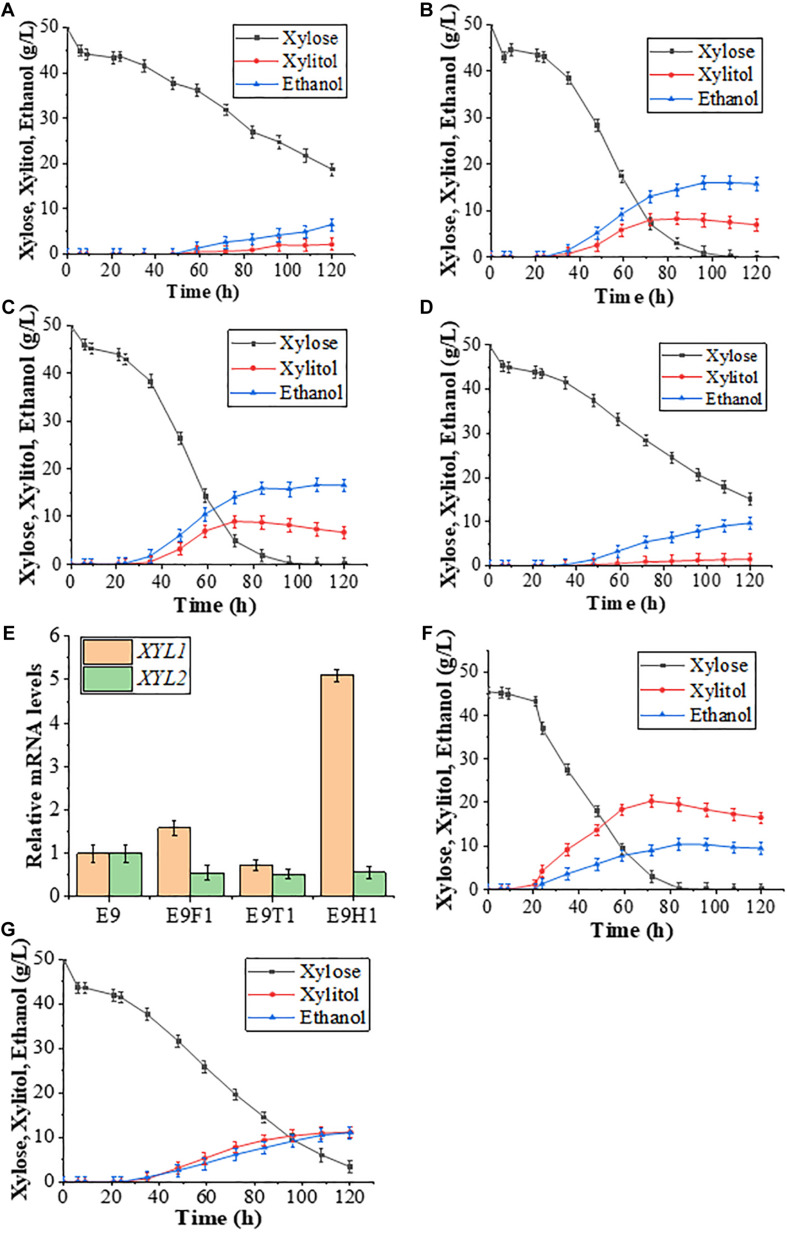
Effects of *XYL1* expression driven by different promoters on the xylose fermentation. **(A–D)** Fermentation results of strains E9T1 **(A)**, E9F1 **(B)**, E9H1 **(C)**, and E9 **(D)** in a YPX medium with 50 g/L xylose are shown. **(E)** Comparison of relative transcription levels of *XYL1* and *XYL2* for strains E9, E9F1, E9T1, and E9H1. **(F,G)** Fermentation results of D9H1 **(F)** and D9 **(G)** in the YPX medium with 50 g/L xylose. Error bars represent standard deviations from three independent experiments.

In order to further improve xylose utilization toward ethanol production, we asked whether proposed rational promoters could replace the original constitutive *ADH1* promoter to effectively drive the *XYL1-K270R* gene. We previously developed a set of *S. cerevisiae* promoters to regulate the xylose metabolic pathway including *HXT7, HXT4, TPI1, FBA1, CCW12, ACO1, HSP26, HSP70, PGK1*, and *ADH1*, resulting in some rational promoter elements like *HXT7* and *FBA1* (Sang et al., 2016). We replaced the *ADH1* promoter with two previously proposed inducible promoters, *HXT7* (Friedrich and Susanne, 2000; Ye et al., 2010) and *FBA1* (Sang et al., 2016), and a newly selected *TEF1*, resulting in strains E9H1, E9F1, and E9T1, respectively. These strains (Figures 2A–C) together with E9 (Figure 2D) were evaluated in the YPX medium to assess effects of *XYL1* expression driven by different promoters on the xylose fermentation, and the results at 120 h are summarized in Table 1. At 120 h, the remaining amount of xylose was 18.73 g/L and the ethanol yield was only 6.50 g/L in E9T1 (Figure 2A). Although accompanied by lower xylitol production, the xylose utilization capacity and ethanol production by E9T1 were not ideal (Figure 2A). In contrast, E9F1 (Figure 2B) and E9H1 (Figure 2C) showed enhanced ethanol production over E9, and E9H1 appears to perform the best among all tested strains. At 120 h, xylose was exhausted in the E9H1 culture, resulting in the production of 16.47 g/L ethanol (Figure 2C), although the xylitol yield was still higher than that in E9 (Figure 2D). The ethanol production of E9F1 was 15.71 g/L, which was slightly lower than that of E9H1 (Table 1). It was speculated that the *HXT7* promoter accelerates the utilization of xylose by expressing higher levels of *XYL1* than the *ADH1* promoter, thereby increasing the ethanol production. However, the xylitol produced by Xyl1-K270R could not be quickly utilized by the downstream XDH, resulting in the xylitol accumulation to a certain extent.

**TABLE 1:** The comparative fermentation performance of xylose, xylitol, and ethanol for different yeast strains E9, E9H1, E9F1, and E9T1 at 120 h.

	Xylose (g/L)	Xylitol (g/L)	Ethanol (g/L)
E9	15.11	1.44	9.64
E9T1	18.73	2.13	6.5
E9F1	0	6.88	15.71
E9H1	0	6.64	16.47

To ask whether the promoter strength indeed plays a critical role in the expression of *XYL1* that leads to the subsequent fermentation performance, we measured relative levels of *XYL1* mRNA in the four engineered strains. The *FBA1* promoter appears to be stronger while the *TEF1* promoter is less effective than the *ADH1* promoter (Figure 2E), which is consistent with our fermentation data. In sharp contrast, the *P_*HXT*__7_-XYL1-K270R* expression is five-fold higher than *P_*ADH*__1_-XYL1-K270R* in the xylose medium (Figure 2E). Despite rather different levels of *XYL1* mRNA in E9F1 and E9H1, their ethanol production was comparable, indicating that the cellular Xyl1 activity is not a sole limiting factor once the *XYL1* gene is overexpressed from the E9 background. Indeed it has been reported that the XR-to-XDH ratio in recombinant *S. cerevisiae* strains affect the xylose utilization (Bao et al., 1997).

To further address roles of *XYL1* promoter strength in the utilization of xylose, we replaced the *ADH1* promoter with the *HXT7* promoter in strain D9 to obtain strain D9H1 and conducted xylose fermentation studies. As expected, the xylose consumption in the D9H1 culture (Figure 2F) was significantly faster than that of D9 (Figure 2G) and E9H1 (Figure 2C), and almost no xylose was observed by 84 h. Consequently, the xylitol yield in the D9H1 (Figure 2F) culture is also much higher than D9 (Figure 2G) and E9 (Figure 2D), reaching a peak of 20.24 g/L by 72 h. The above observations collectively allow us to conclude that increased XR activity can effectively consume xylose, but its product xylitol cannot be further consumed in a timely fashion, resulting in the accumulation of xylitol and compromised ethanol yield under our experimental conditions.

### Combined Ectopic Expression of Key Genes in the PPP Pathway and *XYL2* Facilitates Xylitol Utilization

**FIGURE 3:**
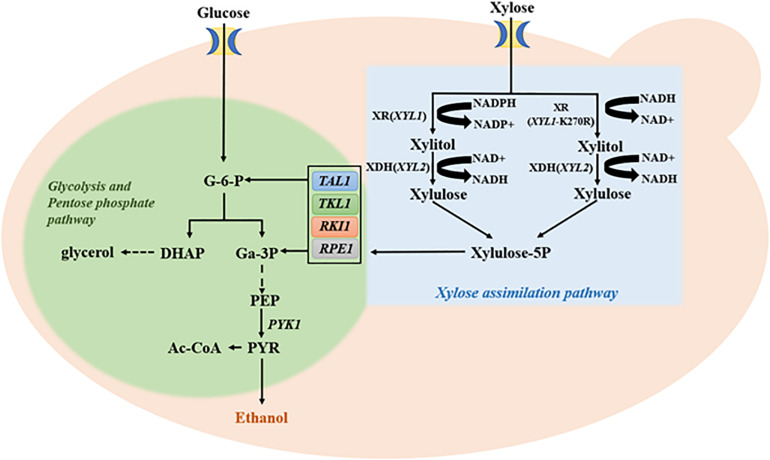
Glucose and xylose metabolic pathways along with key genes and enzymes involved. Abbreviations of related genes: *TAL1*, Transaldolase; *TKL1*, Transketolase; *RPE1*, Ribulose 5-phosphate epimerase; *RKI1*, Ribose 5-phosphate isomerase; *PYK1*, Pyruvate kinase; G-6-P, Glucose-6-phosphate; DHAP, Dihydroxyacetone phosphate; Ga-3P, 3-phosphoglyceraldehyde; PEP, Phosphoenolpyruvate; and PYR, Pyruvate.

**FIGURE 4:**
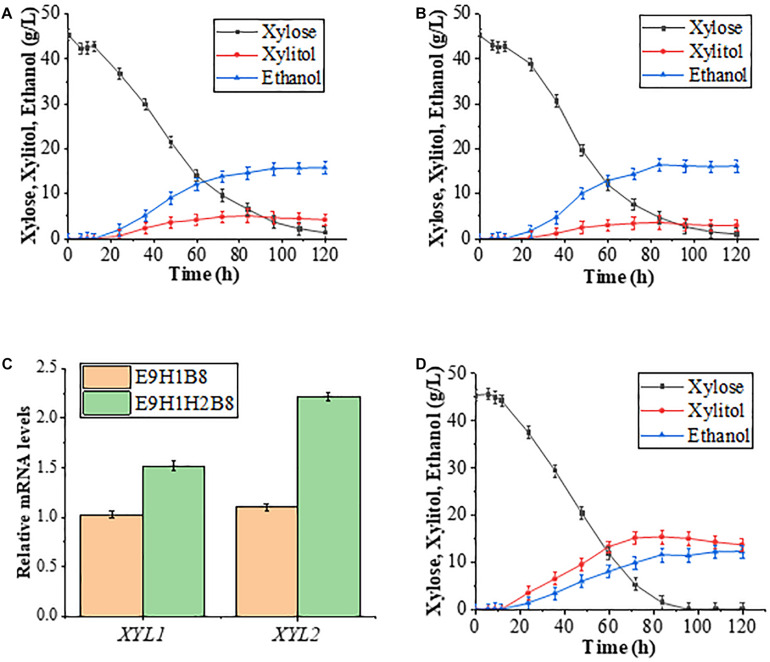
Improvement of xylose fermentation efficiency by altered expression of *XYL2* and key genes in PPP. **(A,B)** Fermentation results of E9H1B8 **(A)** and E9H1H2B8 **(B)** in a YPX medium containing 50 g/L xylose. **(C)** Comparison of relative transcription levels of *XYL1* and *XYL2* in strains E9H1B8 and E9H1H2B8. **(D)** The fermentation performance of D9H1H2B8 was evaluated in a YPX medium containing 50 g/L xylose. Error bars represent standard deviations from three independent experiments.

Effective conversion of xylose into ethanol production requires both upstream xylose utilization and the downstream pentose phosphate pathway (PPP; Figure 3). It has been reported that enhanced PPP by co-expression of four key genes *TAL1*, *TKL1*, *RPE1*, and *RKI1* in PPP promotes the continuous utilization of xylitol and increases ethanol production (Xiong et al., 2011, 2013). Conversely, lack of support by PPP may undermine effects of increased Xyl2. Therefore, this study first introduced plasmid B8 carrying the four PPP genes into E9H1 to form strain E9H1B8 and then evaluated its fermentation in the standard YPX medium. The xylitol production reached 8.91 g/L by 72 h in E9H1 (Figure 2C) and 5.10 g/L by 84 h in E9H1B8 (Figure 4A), and their final xylitol production at 120 h was 6.64 and 4.25 g/L, respectively. Although the overall fermentation performance of E9H1B8 is much better than E9H1, its xylose consumption efficacy remains lower.

To further minimize xylitol accumulation, we replaced the *P_*PGK*__1_-XYL2* promoter with the *HXT7* promoter in strain E9H1B8 to obtain strain E9H1H2B8 and performed the xylose fermentation assay. The xylitol production in the E9H1H2B8 culture reached a peak of 3.59 g/L at 84 h and eventually reduced to 2.95 g/L, while its ethanol production was 16.15 g/L at 120 h (Figure 4B). Hence, compared with E9H1B8, replacing the *XYL2* promoter from *PGK1* to *HXT7* further reduces xylitol content, increases xylose consumption and ethanol production. It is noticed that despite the successful reduction of xylitol by the E9H1H2B8 strain, its ethanol production does not improve over E9H1 (Figures 2C, 4B). We speculate that the metabolic intermediates like xylitol in the E9H1H2B8 were converted to other undetected product(s) or consumed. Future studies could be directed to reduce byproduct(s) and increase ethanol production in strains like E9H1H2B8.

We examined relative expression of *XYL1* and *XYL2* during the logarithmic growth phase in 20 g/L xylose medium and found that the expression of *XYL2* driven by the *HXT7* promoter is more than twofold higher than that by the *PGK1* promoter (Figure 4C), which could explain the enhanced xylose fermentation by strain E9H1H2B8. Surprisingly, the expression of *XYL1* in E9H1H2B8 is 1.5-fold higher than in E9H1B8, although it is driven by the same *HXT7* promoter in both strains.

Although overall xylose fermentation parameters are improved, E9H1B8-based strains could not utilize all xylose in the YPX medium by 120 h. In contrast, D9-based strains appear to consume xylose more efficiently. We asked if introducing B8 and H2 into D9H1 could improve xylose fermentation. As shown in Figure 4D, the xylitol production in the D9H1H2B8 culture was 13.68 g/L, while its ethanol production was 12.27 g/L at 120 h, which is far from desired in comparison to E9H1H2B8 (Figure 4B). Hence, rational expression of XR-K270R was effective in significantly minimizing the xylitol production and converting more carbon resource from xylose to ethanol.

### Regulation of the *HXT7* Promoter by Glucose and Xylose

**FIGURE 5:**
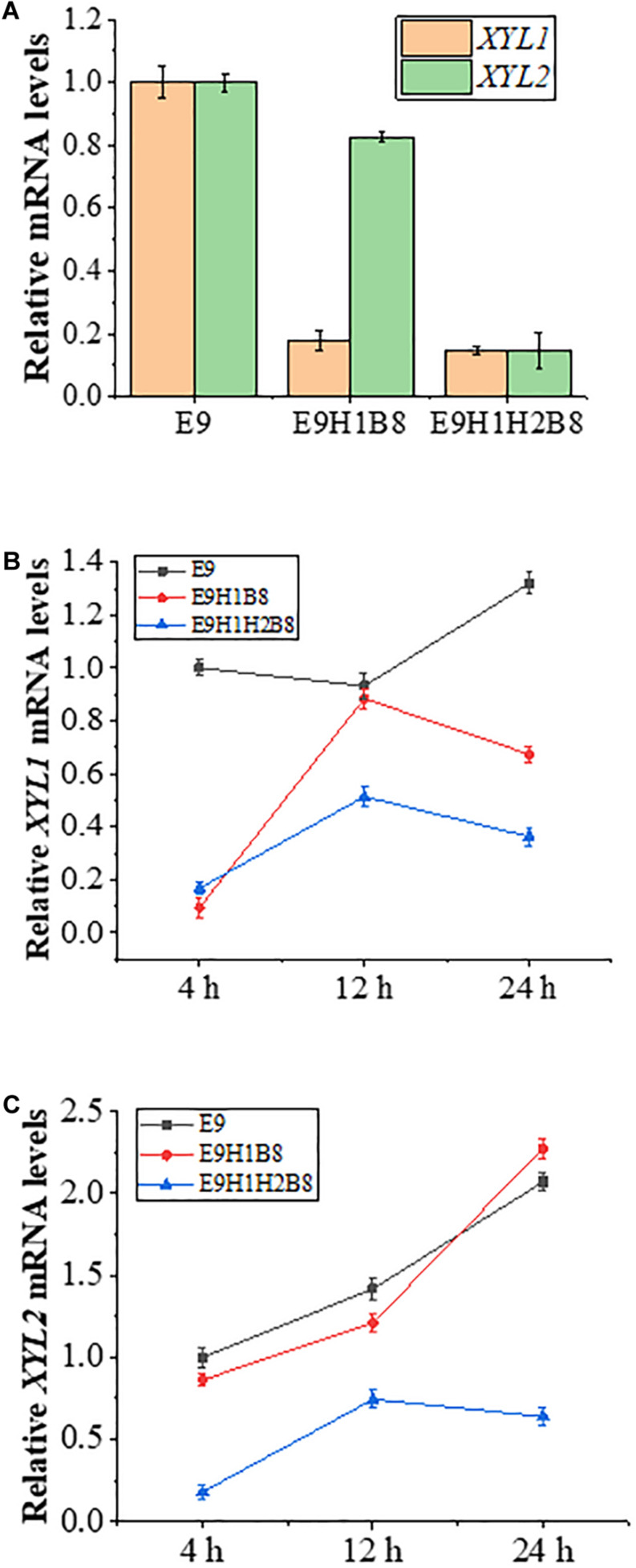
Expression levels of *XYL1* and *XYL2* driven by the *HXT7* promoter under different fermentation conditions. **(A)** Comparison of transcription levels of *XYL1* and *XYL2* in strains E9, E9H1B8, and E9H1H2B8 in a YPX medium containing 20 g/L glucose to logarithmic growth phase. **(B,C)** Comparison of transcription levels of *XYL1* **(B)** and *XYL2* **(C)** for the indicated strains in a YPX medium containing 10 g/L xylose and 10 g/L glucose to logarithmic growth phase. Error bars represent standard deviations from three independent experiments.

To ask how the *HXT7* promoter is regulated in glucose or xylose, we first examined relative expression of *XYL1* and *XYL2* in E9, E9H1B8 and E9H1H2B8 in 20 g/L glucose medium grown to the logarithmic phase, in which *XYL1* is driven by *ADH1*, *HXT7* and *HXT7*, and *XYL2* is driven by *PGK1*, *PGK1*, and *HXT7*, respectively. Figure 5A shows that in the glucose medium, *XYL1* and *XYL2* mRNA levels driven by the *HXT7* promoter are approximately 1/5 of the *ADH1* and *PGK1* promoters, indicating that *HXT7* is repressed in the presence of glucose by up to fivefold. *HXT7* encodes a high-affinity hexose transporter and can be highly expressed under low hexose conditions (Ye et al., 2010). Based on our experimental results, it can be concluded that the *HXT7* promoter is inhibited by glucose and depressed by xylose. When E9-based strains are grown in YPX medium containing 10 g/L glucose and 10 g/L xylose, *S. cerevisiae* preferentially utilizes glucose over xylose so that glucose remains abundant at 4 h and is exhausted by 12 h, while xylose is exhausted by 24 h. As shown in Figures 5B,C, under the above mixed sugar conditions, *ADH1* and *PGK1* promoters remain constitutive, while the *HXT7* promoter is inhibited by glucose and depressed by xylose in the absence of glucose. By 24 h of fermentation, both glucose and xylose were consumed while the *HXT7* promoter remained highly active. Based on the above observations, it is concluded that the *HXT7* promoter is depressed in the xylose medium and is suitable for driving xylose utilization genes to increase ethanol production.

## Conclusion

This study found that although amino acid substitutions in Xyl1 like Xyl1-K270R could balance the redox during xylose fermentation, they may also compromise the enzymatic activity, resulting in decreased xylose utilization. Through optimizing the level of *XYL1-K270R* expression and improving the downstream metabolic capacity, efficient xylose utilization in combination with reduced xylitol accumulation was achieved in both xylose and mixed sugars culture media. It was mainly attributed to the utilization of the *HXT7* promoter, which is found to be repressed in the presence of glucose, depressed by xylose and further induced when the hexose content is low.

## Data Availability Statement

The original contributions presented in the study are included in the article/Supplementary Material, further inquiries can be directed to the corresponding author/s.

## Author Contributions

LC and JZ conceived the idea. YZ and JZ conducted the experiments. YZ, ZJ, and QL analyzed and prepared the data. LC and WX supervised the project and provided financial supports. YZ, LZ, LC, and WX wrote the manuscript. All authors approved the final version.

## Conflict of Interest

The authors declare that the research was conducted in the absence of any commercial or financial relationships that could be construed as a potential conflict of interest.

## Supplementary Material

Supplementary Figure 1 Restriction map of pUC-GU-3X.

Supplementary Table 1 Oligonucleotides used in this study.

Supplementary Table 2 Plasmids used in this study.

Supplementary Table 3 Yeast strains used in this study.
