# Supplementary material for: Minimize the Xylitol Production in Saccharomyces cerevisiae by Balancing the Xylose Redox Metabolic Pathway
Source: Front Bioeng Biotechnol. 2021 Feb 26;9:639595. doi: 10.3389/fbioe.2021.639595 (PMC7953151; doi:10.3389/fbioe.2021.639595)
Supplement: Supplementary Figure 1 — Restriction map of pUC-GU-3X. [file Presentation_1.PPTX]

## Slide 1
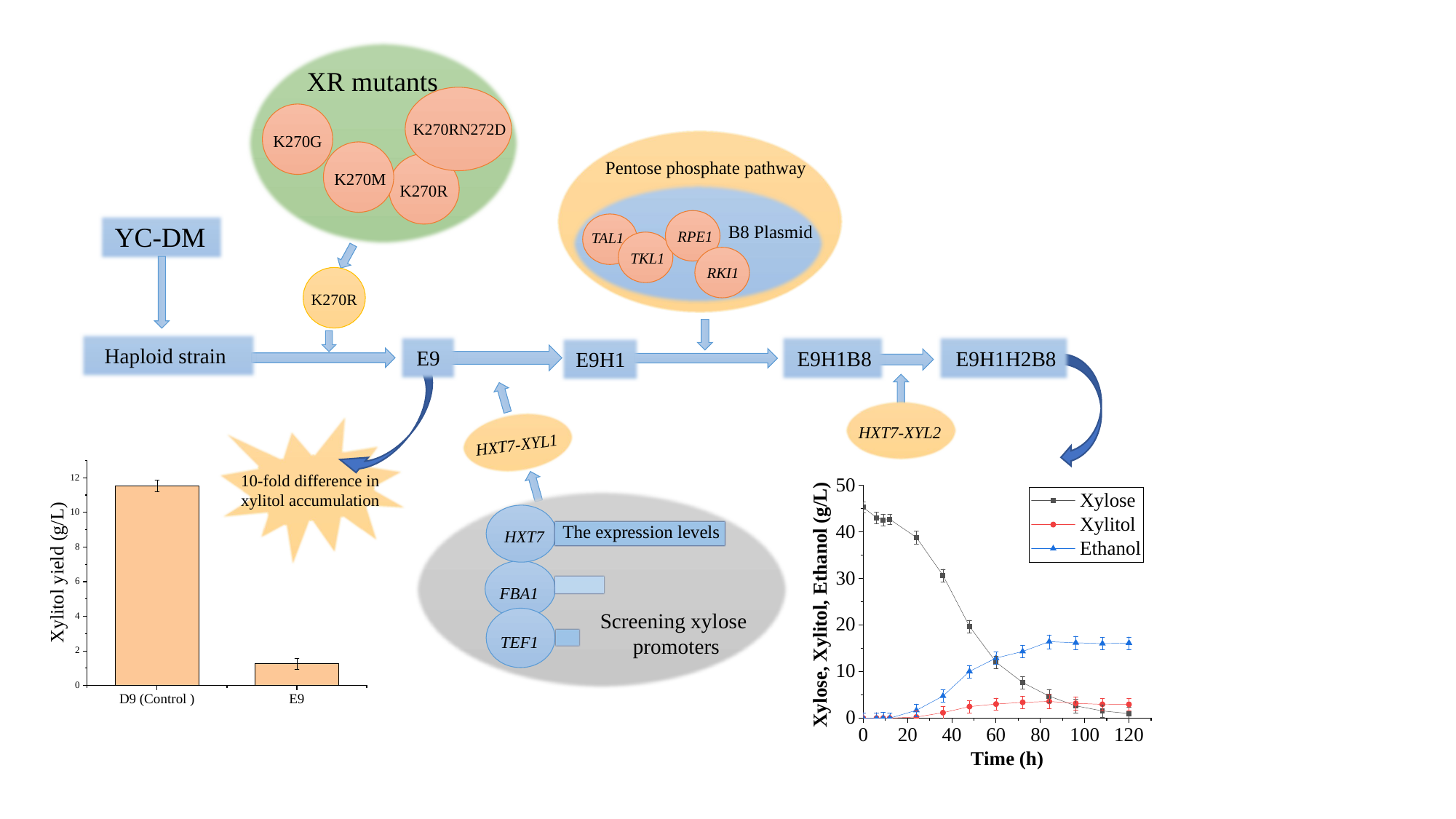

XR mutants
K270RN272D
K270G
K270M
K270R
Pentose phosphate pathway
RPE1
TAL1
B8 Plasmid
TKL1
RKI1
YC-DM
K270R
Haploid strain
E9
E9H1B8
E9H1H2B8
E9H1
HXT7-XYL2
HXT7-XYL1
10-fold difference in xylitol accumulation
HXT7
The expression levels
FBA1
Screening xylose
 promoters
TEF1
