# Supplementary material for: Minimize the Xylitol Production in Saccharomyces cerevisiae by Balancing the Xylose Redox Metabolic Pathway
Source: Front Bioeng Biotechnol. 2021 Feb 26;9:639595. doi: 10.3389/fbioe.2021.639595 (PMC7953151; doi:10.3389/fbioe.2021.639595)
Supplement: Supplementary Table 1 — Oligonucleotides used in this study. [file Data_Sheet_1.docx]

**Supplementary Materials**

Brief description:

Journal: *Frontiers in Bioengineering and Biotechnology*

Title: " **Minimize the xylitol production in *Saccharomyces cerevisiae* by balancing the xylose redox metabolic pathway** "

Authors: Yixuan Zhu, Jingtao Zhang, Lang Zhu, Zefang Jia, Qi Li, Wei Xiao, Limin Cao

The supplementary materials contains three tables and one figure with the figure legend.

**Table S1.** Primers used in this study

| **Primer name** | **Sequence** |
| --- | --- |
| GEA2-F | 5’-TTTGAAACTAGGGAAGACAAGC-3’ |
| PGK1P-R | 5’-ACAAAACCTGTGAGCCGTCG-3’ |
| K270R-F | 5’-GGACAAGATTCCTATCTTCG-3’ |
| URA3-F  URA3-R | 5’-CTAACAATACCTGGGCCCAC-3’  5’-TAACAATACCTGGGCCCACCACAC-3’ |
| HOG1-F | 5’-TACTGTGACGATATCAGCAGCATGC-3’ |
| HOG1-R | 5’-GCCTCGGTGAGTTTTCTCCTTCATT-3’ |
| HOG2-F | 5’-GGAGGCCAAAAATTCATGGAAGAC-3’ |
| HOG2-R | 5’-GGCAATTGCTTCTCTCAAATGCTG-3’ |
| XYL1ORF-F | 5’-CATCAACTTGAGATTCAACGACCC-3’ |
| ADH2p-F | 5’-CCAATGCTCTTATCTATGGGACTTCCGG-3’ |
| ADH2p-R  HXT7p-F  HXT7p-R  GFP-Xma1-F  GFP-Xma1-R  FBA1p-F  FBA1p-R  TEF1p-F  TEF1p-R  XYL1-F  XYL1-R  HXT7p-F  HXT7p-R  Fba1p-F  Fba1p-R  XYL2-F  ADH1-F  ADH1-R  PGK1-F  PGK1-R  HSP26p-BamH1-F  HSP26p-Xma1-R  KGD1p-BamH1-F  KGD1p-Xma1-R  XR-F  XR-R  XDH-F  XDH-R  XKS1-F  XKS1-R | 5’-GGCCCGAAATTGTTCCTACGAGAAGTAG-3’  5’-CGCGGATCCTCTAGTTTCTGCCTTAAA-3’  5’-CGCGGCCCCGGGTTTTTGATTAAAATTAAA-3’  5’-ATTACCCGGGATGGTGAGCAAGGGCGAG-3’  5’-TCCCCCCGGGCTTGTACAGCTCGTCCAT-3’  5’-ATTCATGGATCCAATGCCCTTCATGCCTCC-3’  5’-CCGTCCCCCCGGGTTTGAATATGTATTACTT-3’  5’-GCGCGCGGATCCAAAATGTTTCTACTCCTT-3’  5’-CGCTCCCCCCGGGTTTGTAATTAAAACTTAG-3’  5’-GGGCCCCCCGGGATGCCTTCTATTAAGTTGAAC-3’  5’-GGGCCCCGTACGTTAGACGAAGATAGGAATCT-3’  5’-GCGCGCAAAACTGCAGTCTAGTTTCTGCCTTAAA-3’  5’-GCGCGCGCACGCGTCGACTTTTTGATTAAAATTAAA-3’  5’-AAAACTGCAGAATGCCCTTCATGCCTCC-3’  5’-GCACGCGTCGACTTTGAATATGTATTACTTGG-3’  5’-GGGCCCGTCGACATGACTGCTAACCCTTCCTTGG-3’  5’-GGATCCCAAACCCATACATC-3’  5’-CCCGGGTGTATATGAGATAG-3’  5’-GGGCCCGGATCCAGGCATTTGCAAGAATTACTC-3’  5’-GGGCCCCTGCAGTGTTTTATATTTGTTGTAAAAAGTAG-3’  5’-CGCGGATCCGGGGACCCCAAAAAAATA-3’  5’-TCCCCCCGGGGTTAATTTGTTTAGTTTG-3’  5’-CGCGGATCCACACTCTTCGTAACGTTT-3’  5’-TCCCCCCGGGAACGGTAAAATTTAGTAT-3’  5’-CACGGTAAGTCTCCAGCTCA-3’  5’-TCAGCGAAATCTTGTTCGTC-3’  5’-TAACTCCAAGGAAGGCGAAC-3’  5’-ACCAACAGACAATGGCTCAA-3’  5’-GGGAGATCGTTCCTAGCGTA-3’  5’-CCCTGCAACTTAAAGCCTGT-3’ |

**Table S2. Plasmids used in this study**

| Plasmids | Marker and description | Source |
| --- | --- | --- |
| pUC-GU-3X | Amp^R^, *P_ADH1_-XYL1-T_ADH1_/P_PGK1_-XYL2-T_PGK1_ P_PGK1_-XKS1-T_PGK1_* | (Xiong et al., 2011) |
| pUC-GU-KR-E9 | Amp^R^, *P_ADH1_-XYL1(K270R)-T_ADH1_*/*P_PGK1_-XYL2-T_PGK1_/P_PGK1_-XKS1-T_PGK1_* | (Xiong et al., 2011) |
| B8 | Amp^R^, *P_PDC1_-TKL1-T_TKL1_/P_PGK1_-TAL1-T_TAL1_/P_TPI1_-RKI1-T_RKI1_/P_ADH1_-RPE1-T_RPE1_* | (Xiong et al., 2011) |
| pUC-3XK270M | Amp^R^, *P_ADH1_-XYL1(K270M)-T_ADH1_*/*P_PGK1_-XYL2-T_PGK1_/P_PGK1_-XKS1-T_PGK1_* | (Xiong et al., 2011) |
| pUC-3XK270G | Amp^R^, *P_ADH1_-XYL1(K270G)-T_ADH1_*/*P_PGK1_-XYL2-T_PGK1_/P_PGK1_-XKS1-T_PGK1_* | (Xiong et al., 2011) |
| pUC-3XK270R/N272D | Amp^R^, *P_ADH1_-XYL1(K270RN272D)-T_ADH1_*/*P_PGK1_-XYL2-T_PGK1_/P_PGK1_-XKS1-T_PGK1_* | (Xiong et al., 2011) |
| pUC-P_HXT7_-3XK270R | Amp^R^, *P_HXT7_-XYL1(K270R)-T_ADH1_*/*P_PGK1_-XYL2-T_PGK1_/P_PGK1_-XKS1-T_PGK1_* | This study |
| pUC-P_FBA1_-3XK270R | Amp^R^, *P_FBA1_-XYL1(K270R)-T_ADH1_*/*P_PGK1_-XYL2-T_PGK1_/P_PGK1_-XKS1-T_PGK1_* | This study |
| pUC-P_TEF1_-3XK270R | Amp^R^, *P_TEF1_-XYL1(K270R)-T_ADH1_*/*P_PGK1_-XYL2-T_PGK1_/P_PGK1_-XKS1-T_PGK1_* | This study |
| pUC-P_HXT7_-3X | Amp^R^, *P_HXT7_-XYL1-T_ADH1_/P_PGK1_-XYL2-T_PGK1_ P_PGK1_-XKS1-T_PGK1_* | This study |
| PTZ18R- XYL2 | Amp^R^, *P_PGK1_-XYL2-T_PGK1_* | This study |
| PTZ18R-P_HXT7_-XYL2 | Amp^R^, *P_HXT7_-XYL2-T_PGK1_* | This study |
| PTZ18R-P_FBA1_-XYL2 | Amp^R^, *P_FBA1_-XYL2-T_PGK1_* | This study |
| pUC-P_HXT7_-eGFP-3XK270R | Amp^R^, *P_HXT7_-eGFP-XYL1(K270R)-T_ADH1_/P_PGK1_-XYL2-T_PGK1_/P_PGK1_-XKS1-T_PGK1_* | This study |
| pUC-P_FBA1_-eGFP-3XK270R | Amp^R^, *P_FBA1_-eGFP-XYL1(K270R)-T_ADH1_/P_PGK1_-XYL2-T_PGK1_/P_PGK1_-XKS1-T_PGK1_* | This study |
| pUC-TEF1p-eGFP-3XK270R | Amp^R^, *P_TEF1_-eGFP-XYL1(K270R)-T_ADH1_/P_PGK1_-XYL2-T_PGK1_/P_PGK1_-XKS1-TPGK1* | This study |
| pUC-eGFP-3XK270R | Amp^R^, *P_ADH1_-eGFP-XYL1(K270R)-T_ADH1_/P_PGK1_-XYL2-T_PGK1_/P_PGK1_-XKS1-T_PGK1_* | This study |

Xiong, M., Chen, G., and Barford, J. (2011). Alteration of xylose reductase coenzyme preference to improve ethanol production by Saccharomyces cerevisiae from high xylose concentrations. Bioresource Technology *102*, 9206-9215.

**Table S3. Yeast strains used in this study**

| **Strains** | **Genotype** | **Source** |
| --- | --- | --- |
| YC-DM | *MAT****a****/α* | Angel Yeast, China |
| D9 | *MAT****a*** *P_ADH1_-XYL1-T_ADH1_/P_PGK1_-XYL2-T_PGK1_ P_PGK1_-XKS1-T_PGK1_* | This study |
| E9 | *MAT****a*** *P_ADH1_-XYL1(K270R)-T_ADH1_*/*P_PGK1_-XYL2-T_PGK1_/P_PGK1_-XKS1-T_PGK1_* | This study |
| E6 | *MAT****a*** *P_ADH1_-XYL1(K270G)-T_ADH1_/P_PGK1_-XYL2-T_PGK1_*/*P_PGK1_-XKS1-T_PGK1_* | This study |
| E7 | *MAT****a*** *P_ADH1_-XYL1(K270M)-T_ADH1_*/*P_PGK1_-XYL2-T_PGK1_/P_PGK1_-XKS1-T_PGK1_* | This study |
| E8 | *MAT****a*** *P_ADH1_-XYL1(K270R/N272D)-T_ADH1_/ P_PGK1_-XYL2- T_PGK1_/ P_PGK1_-XKS1- T_PGK1_* | This study |
| B8 | *MAT****a*** *ura3*/*P_PDC1_-TKL1-T_TKL1_/P_PGK1_-TAL1-T_TAL1_/P_TPI1_-RKI1-T_RKI1_/P_ADH1_-RPE1-T_RPE1_* | This study |
| E9H1 | *MAT****a*** *P_HXT7_-XYL1(K270R)-T_ADH1_/**P_PGK1_-XYL2-T_PGK1_/P_PGK1_-XKS1-T_PGK1_* | This study |
| E9F1 | *MAT****a*** *P_FBA1_-XYL1(K270R)-T_ADH1_/P_PGK1_-XYL2-T_PGK1_/P_PGK1_-XKS1-T_PGK1_* | This study |
| E9T1 | *MAT****a*** *P_TEF1_-XYL1(K270R)-T_ADH1_/P_PGK1_-XYL2-T_PGK1_/P_PGK1_-XKS1-T_PGK1_* | This study |
| D9H1 | *MAT****a*** *P_HXT7_-XYL1- T_ADH1_/ P_PGK1_-XYL2- T_PGK1_/ P_PGK1_-XKS1- T_PGK1_* | This study |
| E9H1B8 | *MAT****a*** *P_HXT7_-XYL1(K270R)-T_ADH1_/P_PGK1_-XYL2-T_PGK1_/P_PGK1_-XKS1-T_PGK1_/P_PDC1_-TKL1-T_TKL1_/P_PGK1_-TAL1-T_TAL1_/P_TPI1_-RKI1-T_RKI1_/P_ADH1_-RPE1-T_RPE1_* | This study |
| D9H2B8 | *MAT****a*** *P_ADH1_-XYL1-T_ADH1_/P_HXT7_-XYL2-T_PGK1_*/*P_PGK1_-XKS1-T_PGK1_/P_PDC1_-TKL1-T_TKL1_/P_PGK1_-TAL1-T_TAL1_/P_TPI1_-RKI1-T_RKI1_/P_ADH1_-RPE1-T_RPE1_* | This study |
| D9H1H2B8 | *MAT****a*** *P_HXT7_-XYL1-T_ADH1_/P_HXT7_-XYL2-T_PGK1_/P_PGK1_-XKS1-T_PGK1_/P_PDC1_-TKL1-T_TKL1_/P_PGK1_-TAL1-T_TAL1_/P_TPI1_-RKI1-T_RKI1_/P_ADH1_-RPE1-T_RPE1_* | This study |
| E9H1H2B8 | *MAT****a*** *P_HXT7_-XYL1*(K270R)-*T_ADH1_/P_HXT7_-XYL2-T_PGK1_/P_PGK1_-XKS1-T_PGK1_/P_PDC1_-TKL1-T_TKL1_/P_PGK1_-TAL1-T_TAL1_/P_TPI1_-RKI1-T_RKI1_/P_ADH1_-RPE1-T_RPE1_* | This study |

**Figure S1:** A restriction map of pUC-GU-3X. *XYL1* is driven by the *ADH1* promoter, while *XKS1* and *XYL2* are driven by the *PGK1* promoter.

**
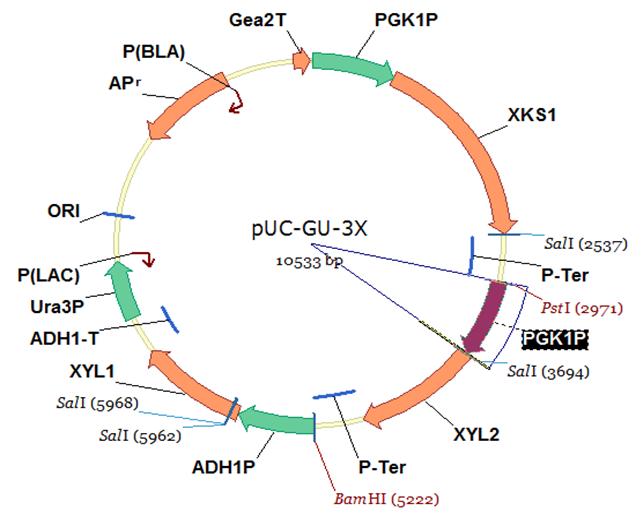
**
